# Examining the role of biologic sex on kidney outcomes in preterm neonates: A secondary analysis of the PENUT/REPAIReD study

**DOI:** 10.1007/s00467-025-07131-3

**Published:** 2026-03-12

**Authors:** Michelle C. Starr, Russell Griffin, Heidi J. Steflik, Krithika Lingappan, Matt Gillen, David T. Selewski, David J. Askenazi, Shina Menon, Cara L. Slagle, Danielle E. Soranno

**Affiliations:** 1 Division of Pediatric Nephrology, Department of Pediatrics, Indiana University School of Medicine, 410 W 10 Street, Suite 2000A, Indianapolis, IN 46202 USA; 2 Division of Child Health Service Research, Department of Pediatrics, Indiana University School of Medicine, 410 W 10th Street, Suite 2000A, Indianapolis, IN 46202 USA; 3 Department of Epidemiology, University of Alabama at Birmingham, Birmingham, AL USA; 4 Division of Neonatal-Perinatal Medicine, Department of Pediatrics, Medical University of South Carolina, Charleston, SC USA; 5 Division of Neonatology, Children’s Hospital of Philadelphia, University of Pennsylvania Perelman School of Medicine, Philadelphia, PA USA; 6 Division of Pediatric Nephrology, Emory University School of Medicine, Atlanta, GA USA; 7 Division of Pediatric Nephrology, Department of Pediatrics, University of Arkansas for Medical Sciences, Little Rock, AR USA; 8 Division of Pediatric Nephrology, Department of Pediatrics, University of Alabama at Birmingham, Birmingham, AL USA; 9 Division of Pediatric Nephrology, Department of Pediatrics, Stanford University, Palo Alto, CA USA; 10 Division of Perinatal Medicine, Department of Pediatrics, Indiana University School of Medicine, Indianapolis, IN USA; 11 Weldon School of Biomedical Engineering, Purdue University, West Lafayette, IN USA

**Keywords:** Acute kidney injury, Chronic lung disease, Chronic kidney disease, Hypertension, Prematurity

## Abstract

**Background:**

Biological sex plays a crucial role in the pathophysiology of morbidities related to preterm birth. Studies specifically investigating the role of biological sex in neonatal kidney disease are lacking. This study aimed to determine the association between biological sex and kidney outcomes in preterm infants. We hypothesized that male infants would be more likely to have poor kidney outcomes, including acute kidney injury (AKI), hypertension and chronic kidney disease. Given data on the relationship between sex, AKI and lung disease, we also evaluated lung disease as a secondary outcome.

**Methods:**

Retrospective analysis of the Preterm Erythropoietin Neuroprotection Trial data. For adjusted models, we used covariates associated with adverse outcomes a priori (gestational age, SGA status) and a lasso regression. AKI was defined using creatinine only neonatal modified KDIGO criteria and bronchopulmonary dysplasia (BPD) by Neonatal Research Network criteria.

**Results:**

Of the 923 infants included, 479 (51.8%) were male. AKI was more common among males (aOR 1.31, 95%CI 1.00–1.74). Hypertension was nearly twice as common in males (66.4% vs. 51.5%, *p* < 0.001) and remained after adjustment (aOR 1.91, 95%CI 1.32–2.77). Severe BPD was more common in males (33.4% vs. 24.3%, *p* = 0.0024), which persisted after adjustment (aOR 1.65, 95%CI 1.22–2.23). This association was not fully mitigated by AKI exposure.

**Conclusions:**

We describe differences in kidney outcomes and BPD by sex. Male sex is associated with an increased risk of AKI and hypertension. Research efforts focused on the mechanisms underlying sex-specific differences are needed for the identification of novel therapies that benefit both sexes.

**Graphical abstract:**

**Figure:**
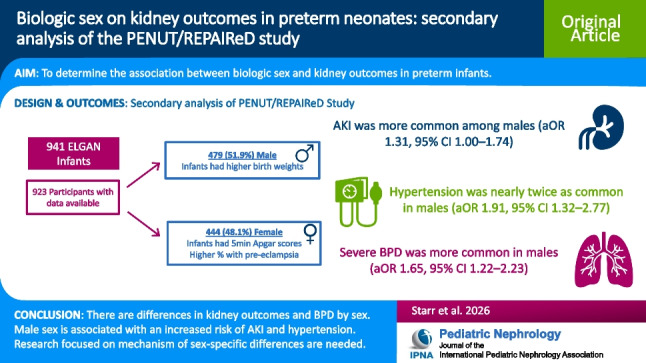
A higher-resolution version of the Graphical abstract is available as  Supplementary information

**Supplementary Information:**

The online version contains supplementary material available at 10.1007/s00467-025-07131-3.

## Introduction

Sex is an important biological variable in the development of multiple diseases in preterm neonates. The evidence of the role of sex as a biological variable in pathophysiology, disease outcome, and response to therapy in neonatal clinical studies continues to increase [1–3]. This includes the development of bronchopulmonary dysplasia (BPD) and intraventricular hemorrhage (IVH) [4, 5]. In older populations, sex has been found to be an important biological variable for kidney-specific outcomes such as acute kidney injury (AKI) and progression to chronic kidney disease [6–11]. For example, female sex is protective in ischemia–reperfusion AKI and deleterious in some forms of nephrotoxin-mediated AKI. Possible explanations for these findings include hormonal differences (which may be mitigated in the perinatal period by circulating maternal hormones) and chromosomal differences (XX vs. XY complement).

Studies investigating the role of biological sex in neonatal kidney diseases, including AKI, are lacking. Most clinical studies do not report data disaggregated by sex. The National Institutes of Health published a call to action for the inclusion of both sexes in pre-clinical and clinical research to increase the transparency of studies [6]. Determining if sex-based differences exist and the mechanism of protective sex biases would allow researchers to identify therapeutic targets that benefit both sexes and has recently been highlighted as an important area of further research [12].

We performed a secondary analysis of the Preterm Erythropoietin Neuroprotection (PENUT) Trial, a multicenter clinical trial that randomized extremely preterm neonates to receive erythropoietin or placebo for neuroprotection. The PENUT study captured robust data including short and long-term kidney-related outcomes [13–15]. While several secondary analyses of the PENUT study have previously reported individual morbidities including AKI, BPD, and late-onset infections, this present study extends this body of work by evaluating sex as a primary exposure across multiple organ systems [14, 16, 17]. We sought to determine the association between biological sex and short-term and long-term kidney outcomes in preterm infants. This integrated approach allows assessment of the association between biologic sex and outcomes within the same cohort with harmonized methods. We hypothesized that male infants would be more likely to have poor short-term and long-term kidney outcomes. Given existing studies that have found a strong relationship between sex and AKI as well as AKI and BPD, we also hypothesized that AKI would mitigate the long-established relationship between sex and BPD [18–20].

## Methods

### Study design

The PENUT Trial was a Phase III randomized, placebo-controlled, double-blind trial of erythropoietin in extremely preterm neonates in 19 academic centers in the United States [15]. The inclusion criteria were (1) gestational age 24 0/7 and 27 6/7 weeks’, (2) enrollment < 24 h of age, and (3) arterial or venous access. Exclusion criteria included (1) major life-threatening anomalies, (2) hematologic crises (e.g., disseminated intravascular coagulation, hemolysis), (3) hematocrit > 65%, (4) hydrops fetalis, and (5) congenital infection.

The Recombinant Erythropoietin for Protection of Infant Renal Disease (REPAIReD) Study was an ancillary study to assess longitudinal kidney function. The Institutional Review Board (IRB) at the University of Washington served as the central IRB and each center involved in the study received approval from their IRB or Human Research Ethics Committee. Consent was obtained from the parent or legal guardian. The PENUT trial is registered with ClinicalTrials.org (NCT01378273). This was a post hoc analysis performed in March 2025 and follows the STROBE reporting guidelines for observational studies [21].

### Definitions of exposure and key outcomes

Sex was determined for each study participant by chart review of the medical record, with this assigned as either male, female, or unknown. We determined AKI stage using Kidney Diseases Improving Global Outcomes definition [22]. Severe AKI was defined as Stage 2 or 3. We did not use urine output criteria to determine AKI. For AKI determination, we used a baseline of the lowest creatinine at any point starting on postnatal day 2. Individual creatinine measurements had to be > 0.5 mg/dL to be considered in the AKI calculation. As creatinine levels on postnatal days 0–2 may represent maternal levels, we began AKI calculation on postnatal day 3 [13]. We note that our AKI definition used all creatinine values collected per protocol, which differs from Turner et al., who excluded infants without daily creatinine available and applied slightly different criteria in AKI definitions [17].

We determined BPD severity by respiratory support at 36 weeks’ post-menstrual. We evaluated a composite outcome of severe BPD or death, defined using the most recent Neonatal Research Network definitions [23]. We used severe BPD (Neonatal Research Network defined Grade 2 or Grade 3 BPD) to focus on preterm neonates most at risk for long-term pulmonary disease [23, 24]. We used a composite of severe BPD/death given that they are competing outcomes. Infants without complete respiratory data or without creatinine measurements were excluded, resulting in a slightly narrower analytic cohort than previous BPD studies in this cohort [16].

For two-year outcomes, we included those with estimated glomerular filtration rate (eGFR), urine albumin/creatinine ratio (ACR), or blood pressure (BP) measured at the two-year visit. We excluded those with death prior to 24 months, no follow-up, no blood or urine samples obtained, nor BP measured. Of the 780 infants with 2-year data, 565 had at least one kidney outcome measure including 348 with eGFR, 435 with urine testing, and 289 with BP measurement [25]. We evaluated three a priori designated kidney outcomes at two years [20, 27]: (1) eGFR < 90 ml/min/1.73 m^2^ using the CKID U25 equation (including sCr and cystatin C) [28, 29], (2) albuminuria, defined as ACR > 30 mg albumin/g creatinine [15], and (3) systolic or diastolic BP > 90th percentile for age and sex based on 2017 AAP guidelines (i.e., hypertension) [27, 30]. Urine samples were collected using a bag specimen or a cotton ball in the diaper. BP readings were obtained using a Briggs Mabic Healthcare Manual Sphygmomanometer with an appropriately sized BP cuff, measured twice, with a five-minute interval between readings. The lowest systolic BP and diastolic BP were recorded [20, 21].

### Statistical analysis

Categorical variables were analyzed by proportional differences with the chi-square test or the Fisher exact test. *T*-test and Wilcoxon rank-sum test were used to compare continuous and ordinal variables, respectively. Odds ratios (OR) and associated 95% confidence intervals (CIs) for the association between sex and outcomes of interest were estimated from unconditional logistic regression models. Given the existing studies from our group and others that have found a strong relationship between sex and AKI as well as AKI and BPD, and a concern that AKI may play a role in this relationship between sex and the composite outcome, we then stratified our multivariable analysis by AKI status [18–20].

Multivariable logistic regression was performed using a lasso regression model. We created three multivariable models in keeping with existing literature evaluating sex differences in neonatal outcomes. Our models included one adjusted for gestational age, one by gestational age and small for gestational age (SGA) status, and then a final model including an a priori list of variables (gestational age, small for gestational age (SGA) status, maternal race, pre-eclampsia, intubation, surfactant use, and chest compressions during resuscitation) based on the existing literature was used to account for potential confounding variables. All findings are reported as adjusted ORs with 95%CIs. In all analyses, a *p*-value < 0.05 was considered statistically significant. Analysis was performed using SAS version 9.4 (Statistical Analysis Software Institute Inc., Cary, NC, USA).

## Results

Of the 941 neonates who met inclusion criteria for PENUT, 923 neonates were eligible for this analysis. The reasons for exclusion of the 18 neonates included no treatment given in the parent trial (*n* = 4), the patient being incorrectly enrolled (*n* = 1) or death within the first two days of life (*n* = 13), all of which were exclusion criteria in the parent trial [15]. Of those included in this analysis, 51.9% (479) were male and 48.1% (444) were female (Table 1). The median birth weight was 800 g (IQR 668, 940), and 15.3% were SGA. Maternal comorbidities were common in the cohort, including multiple gestations (26.4%), pre-eclampsia (15.2%), and hypertension (7.5%). Antenatal steroids were received by 91.6% of mothers of infants in the study. Most infants (97.2%) required some delivery room resuscitation, with 81.0% intubated in the delivery room.

**Table 1:** Baseline neonatal and maternal characteristics of cohort stratified by infant sex, male vs. female

Characteristic	Overall (*n* = 923)	Male infants (*n* = 479)	Female infants (*n* = 444)	*p*-value*
Erythropoietin (%)	454 (49.2)	228 (47.6)	226 (50.9)	0.32
Gestational age, weeks (%)				
24	227 (24.6)	121 (25.3)	106 (23.9)	0.94
25	242 (26.2)	122 (25.5)	120 (27.0)	
26	220 (23.8)	114 (23.8)	106 (23.9)	
27	234 (25.4)	122 (25.5)	112 (25.2)	
Median Birth weight, grams (IQR)	800 (668–940)	820 (690–953)	770 (641–900)	< 0.0001
Small size for gestational age (%)	141 (15.3)	65 (13.7)	76 (17.2)	0.14
Median Apgar 1 min (IQR)	4 (2–6)	4 (2–6)	4 (2–6)	0.07
Median Apgar 5 min (IQR)	7 (5–8)	7 (5–8)	7 (5–8)	0.02
Delivery room resuscitation (%)				
Any	896 (97.2)	469 (97.9)	427 (96.4)	0.16
Intubation	748 (81.0)	396 (82.7)	352 (79.3)	0.19
Surfactant	480 (52.0)	254 (53.0)	226 (50.9)	0.52
Chest compressions	72 (7.8)	34 (7.1)	38 (8.6)	0.41
Resuscitation drugs	32 (3.4)	19 (3.9)	13 (2.9)	0.39
Maternal characteristics (%)				
Multiple gestations	243 (26.4)	126 (26.3)	117 (26.4)	0.99
Diabetes	48 (5.2)	27 (5.6)	21 (4.7)	0.54
Hypertension	70 (7.5)	29 (6.0)	41 (9.2)	0.07
Pre-eclampsia	140 (15.2)	58 (12.1)	82 (18.5)	0.007
Antenatal steroids	831 (91.6)	430 (91.9)	401 (91.3)	0.77
Maternal race (%)				
Black	239 (25.9)	115 (24.0)	124 (27.9)	0.38
White	604 (65.4)	320 (66.8)	284 (64.0)	
Other/Unknown	80 (8.7)	44 (9.2)	36 (8.1)	
Maternal ethnicity (%)				
Hispanic or Latino	197 (21.2)	105 (21.6)	92 (20.7)	0.83
Not Hispanic or Latino	715 (77.5)	369 (77.0)	346 (77.9)	
Unknown	11 (1.2)	5 (1.0)	6 (1.4)	

*Estimated from a chi-square or Wilcoxon Rank sums test for categorical and continuous variables, respectively

Male infants had higher median birth weights (820 g vs. 770 g, *p* < 0.0001), while female infants had higher 5-min Apgar scores (*p* = 0.02). A higher proportion of mothers of female infants had pre-eclampsia (18.5% vs. 12.1%, *p* = 0.007). Other maternal characteristics, including race, ethnicity, and maternal co-morbidities, did not differ significantly by infant sex.

### Short-term outcomes

Rates of AKI of any stage were higher among males (43.8%) than females (37.4%, *p* = 0.046); however, there was no difference in the rate of severe AKI between males and females (Table 2). In fully adjusted models, a similar association was observed between biologic sex and AKI, though the association was only significant for any stage AKI; specifically, males had 30% higher odds of any stage AKI (OR 1.31, 95%CI 1.00–1.74). The adjusted association was not seen for severe AKI (OR 1.33, 95%CI 0.95–1.85) (Table 3).

**Table 2:** In-hospital and long-term outcomes stratified by infant sex, male vs. female

In-hospital outcomes	Overall (*n* = 923)	Male infants (*n* = 479)	Female infants (*n* = 444)	*p*-values*
Short-term, *n* (%)				
AKI				
Any stage	376 (40.7)	210 (43.8)	166 (37.4)	0.0462
Stage 2/3	196 (21.2)	112 (23.4)	84 (18.9)	0.0976
PDA (treated)	389 (42.0)	212 (43.9)	177 (39.9)	0.2145
Death	100 (10.8)	56 (11.7)	44 (9.9)	0.3844
Respiratory, *n* (%)				
Mechanical Ventilation at 14 postnatal days	480 (52.0)	260 (54.3)	220 (49.5)	0.1507
Severe BPD	268 (29.0)	160 (33.4)	108 (24.3)	0.0024
Death or Severe BPD	341 (36.9)	199 (41.5)	142 (32.0)	0.0026
Respiratory status at discharge				
Room air	510 (55.7)	264 (55.2)	246 (56.2)	0.3131
Nasal cannula	306 (33.2)	154 (32.2)	152 (34.7)	
Tracheostomy	14 (1.5)	7 (1.5)	7 (1.6)	
Ventilated	86 (9.3)	53 (11.1)	33 (7.5)	
IVH				
Severe IVH	122 (13.1)	68 (14.0)	64 (12.2)	0.3619
PVL	55 (5.9)	32 (6.6)	23 (5.2)	0.3360
ROP	60 (6.5)	33 (6.8)	27 (6.1)	0.6187
NEC	99 (10.7)	52 (10.7)	47 (10.6)	0.9039
Median Length of stay (IQR)	93 (71–114)	93 (71–118)	92 (71–111)	0.3404
Long-term				
Low eGFR	54/336 (16.1)	28/175 (16.0)	26/161 (16.1)	0.9704
Albuminuria	155/435 (35.6)	74/233 (31.8)	81/202 (40.1)	0.0701
Hypertension	290/492 (58.9)	166/251 (66.1)	124/241 (51.5)	0.0009

^*^Estimated from a chi-square or Wilcoxon rank sums for categorical and continuous variables, respectively

*AKI*, Acute Kidney Injury; *BPD*, Bronchopulmonary Dysplasia; *eGFR*, estimated Glomerular Filtration Rate; *IQR*, Interquartile Range; *IVH*, Intraventricular Hemorrhage; *NEC*, Necrotizing Enterocolitis; *PDA*, Patent Ductus Arteriosis; *PVL*, Periventricular Leukomalacia; *ROP*, Retinopathy of Prematurity

**Table 3:** Multivariable analysis of kidney outcomes in male and female infants

Variable	Male infant	Female infant	Model 1^1,2^	Model 2	Model 3	Model 4
Kidney outcomes						
AKI						
Any stage	210/479 (43.8)	166/444 (37.4)	1.31 (1.00–1.70)	1.32 (1.01–1.74)	1.34 (1.01–1.76)	1.31 (1.00–1.74)
Stage 2/3	112/479 (23.4)	84/444 (18.9)	1.31 (0.95–1.80)	1.31 (0.95–1.81)	1.35 (0.97–1.88)	1.33 (0.95–1.85)
Low eGFR	28/175 (16.0)	26/161 (16.1)	0.99 (0.55–1.77)	0.98 (0.54–1.76)	0.98 (0.54–1.79)	1.07 (0.58–1.98)
Albuminuria	74/233 (31.8)	81/202 (40.1)	0.69 (0.46–1.02)	0.68 (0.46–1.01)	0.70 (0.47–1.04)	0.68 (0.45–1.04)
Hypertension	166/251 (66.1)	124/241 (51.5)	1.84 (1.28–2.65)	1.86 (1.29–2.69)	1.86 (1.29–2.69)	1.91 (1.32–2.77)
Pulmonary outcomes						
Death/Severe BPD	199 (41.5)	142 (32.0)	1.52 (1.16–1.99)	1.54 (1.17–2.02)	1.60 (1.21–2.11)	1.65 (1.24–2.19)
Death	56 (11.7)	44 (9.9)	1.19 (0.78–1.80)	1.20 (0.78–1.82)	1.26 (0.82–1.94)	1.31 (0.84–2.02)
Severe BPD	160 (33.4)	108 (24.3)	1.56 (1.17–2.08)	1.56 (1.17–2.09)	1.62 (1.20–2.17)	1.65 (1.22–2.23)
Pulmonary outcomes stratified by AKI status						
No AKI	269	278				
Death/Severe BPD	100 (37.2)	78 (28.1)	1.52 (1.06–2.18)	1.56 (1.09–2.25)	1.59 (1.10–2.30)	1.70 (1.17–2.48)
Death	27 (10.0)	22 (7.9)	1.27 (0.71–2.29)	1.32 (0.73–2.39)	1.33 (0.73–2.42)	1.38 (0.75–2.55)
Severe BPD	76 (28.3)	58 (20.9)	1.51 (1.02–2.23)	1.53 (1.03–2.26)	1.54 (1.04–2.29)	1.62 (1.08–2.42)
AKI	210	166				
Death/Severe BPD	99 (47.1)	64 (38.6)	1.44 (0.95–2.17)	1.45 (0.95–2.20)	1.59 (1.04–2.44)	1.59 (1.03–2.45)
Death	29 (13.8)	22 (13.3)	1.04 (0.57–1.89)	1.05 (0.57–1.91)	1.17 (0.64–2.15)	1.21 (0.65–2.26)
Severe BPD	84 (40.0)	50 (30.1)	1.57 (1.02–2.41)	1.57 (1.02–2.42)	1.68 (1.08–2.61)	1.68 (1.07–2.63)

^1^Estimated from a logistic regression

^2^Models: 1. Unadjusted OR (95%CI), 2. Adjusted for gestational age, 3. Adjusted for gestational age and SGA status, 4. Adjusted for gestational age, SGA status, maternal race, pre-eclampsia, and use intubation, surfactant use, and chest compressions during resuscitation

Non-kidney short-term complications such as treated patent ductus arteriosus, need for mechanical ventilation at 14 days, and respiratory support at discharge did not differ significantly by sex, though trends showed slightly higher rates of complications in males (Table 3). Rates of severe IVH, periventricular leukomalacia, retinopathy of prematurity, and necrotizing enterocolitis were comparable between groups, and length of hospital stay was nearly identical (median of approximately 93 days with an IQR of 71 to 114 days).

### Long-term outcomes

The rates of low eGFR and albuminuria at two years were similar between sexes, with no differences in prevalences. Males had lower rates of albuminuria at 2 years (31.8% vs. 40.1%); however, this finding was not significant in either unadjusted or adjusted models (Table 3).

Hypertension was significantly more common in male infants (66.1%) compared to females (51.5%, *p* < 0.001). This association remained in the multivariable models, with males being almost twice as likely to have hypertension at two years of age than females (aOR 1.9, 95%CI 1.32–2.77) (Table 3).

### Evaluating the relationship between AKI and BPD

Among the cohort, 100 (10.8%) died and 268 (29.0%) had severe BPD diagnosed at 36 weeks’ postmenstrual age, with 341 (36.9%) meeting the composite outcome of death or severe BPD (Table 2). While overall mortality was similar between male and female infants (11.7% vs. 9.9%, *p* = 0.38), male infants had a higher incidence of severe BPD at 33.4% compared to 24.3% in females (*p* = 0.0024). When combining death or severe BPD as a composite outcome, male infants had worse outcomes (41.5% vs. 32.0%, *p* = 0.0026), indicating a higher burden of severe respiratory morbidity among male preterm infants. In fully adjusted models, males were 65% more likely to experience the composite outcome of death or severe BPD (OR 1.65, 95%CI 1.24–2.19) and approximately 1.7 times more likely to develop severe BPD (OR 1.65, 95%CI 1.22–2.23) (Table 3). There was no difference by AKI status, in fully adjusted models; in particular, the association between male sex and severe BPD was OR 1.62 (95%CI 1.08–2.42) among neonates without AKI and 1.68 (95%CI 1.07–2.63) for neonates with AKI, and for the combined outcome of death and severe BPD was OR 1.70 (95%CI 1.17–2.48) among neonates without AKI and OR 1.59 (95%CI 1.03–2.45) for neonates with AKI (Table 3).

## Discussion

In this large multicenter secondary analysis of preterm infants, we found that male infants exhibited higher rates of AKI and hypertension by 2 years of age. Although prior secondary analysis of the PENUT cohort have characterized individual morbidities, our findings extend this body of work by evaluating sex as the primary exposure using harmonized analytic methods across outcomes. Male sex remained independently associated with a greater risk of AKI during the neonatal period, and the relationship between male sex and respiratory outcomes persisted after stratifying by AKI status. Thus, our findings indicate a male disadvantage across respiratory and kidney morbidity in this extremely preterm cohort.

Our results align with a growing body of literature on sex-related disparities in neonatal outcomes [26]. Previous studies have repeatedly demonstrated that male preterm infants experience worse respiratory outcomes [1]. For example, a propensity-matched analysis found that extremely low birth weight males had a significantly higher incidence of respiratory distress syndrome and moderate-to-severe BPD after adjustment for confounders [11]. Likewise, male infants require more invasive respiratory support and are more likely to need home oxygen or tracheostomy when diagnosed with BPD [27]. We note that mothers of female infants were more likely to have pre-eclampsia, which has been found to have a maturational impact on the lungs of infants. This theoretically would lead to a decrease in lung disease in female infants and therefore a reduced rate of BPD — however, we adjusted for pre-eclampsia in our analysis and the finding that male infants had a higher rate of BPD persisted. Our finding of increased severe BPD and adverse composite outcomes in males is therefore consistent with this “male disadvantage” in neonatal respiratory disease.

Data about kidney outcomes are more limited but suggest a similar pattern of vulnerability [6]. Clinical reports have noted a male predominance among neonatal AKI cases [13, 14]. Experimental models support the idea that female sex is relatively protective: in an animal model of neonatal nephron loss, only male rats developed severe long-term kidney injury, leading the authors to conclude that male sex may be an independent risk factor for kidney disease [28]. These observations lend context to our finding that, even after adjusting for gestational age and other factors, male infants were at higher risk for AKI. We note that while several of our findings reached statistical significance, the absolute effect sizes were modest. These findings align with well-described male disadvantage in neonatal outcome but also suggest that sex, while biologically relevant, likely serves as one of multiple factors contributing to risk stratification.

Several findings from our study are novel. The most striking is the rate of hypertension among male infants at 2 years. While prematurity itself has been found to be a risk factor for childhood hypertension, few prior studies have reported sex differences in this outcome. In the PENUT cohort as a whole, nearly one-quarter of infants had systolic blood pressure above the 95th percentile by 2 years; our secondary analysis suggests that this burden falls disproportionately on males [25]. The mechanisms behind this sex-specific risk for hypertension remain unclear, but can be hypothesized to be in part due to differences in renal and cardiovascular development. Male neonates might be more vulnerable to nephron injury or have different renin–angiotensin and androgenic influences than females, leading to higher blood pressure [28]. Likewise, our finding of an adjusted association between male sex and AKI suggests a potential sex-based susceptibility. This may reflect sex differences in kidney maturation or response to injury; for instance, female preterm infants have been reported to have higher antioxidant enzyme activity than males, which could mitigate injury [2]. Regardless of mechanism, the clinical importance is clear: male preterm infants in our study not only fared worse in the neonatal intensive care unit but also carried more evidence of kidney and cardiovascular sequelae into early childhood.

Our study has notable strengths. The use of data from the PENUT/REPAIReD cohort has robust follow-up and prospectively collected data on respiratory, neurologic, and renal outcomes through 2 years, allowing comparisons by sex. Our analysis was able to adjust for many relevant covariates, lending confidence that observed sex differences are not solely due to measured confounders. However, there are also limitations. This is a secondary observational analysis, so causality cannot be established. Residual confounding by unmeasured variables (e.g., differential exposure to nephrotoxins or ventilation practices by sex) remains possible. Our definition of AKI relied on serum creatinine, which is imperfect in neonates, and misclassification bias may have occurred. Furthermore, we did not evaluate the timing of AKI event. Given this, we were unable to fully capture the temporality of relationship between AKI and other complications (for example, PDA). This limitation may have resulted in misclassification of early vs. late exposures. We also note that a recent analysis in this cohort reported no sex differences in AKI incidence [17]. However, differences between studies include variations in inclusion criteria, the timing and frequency of creatinine values included, and the analytic handling of missing data. Our analysis applied a stricter KDIGO-based definition with broader creatinine capture and included more infants in the PENUT cohort based on the analytical questions evaluated, which may increase sensitivity of mild AKI diagnosis. These methodologic differences may account for our divergent findings. Additionally, blood pressure measurements in toddlers can be variable and influenced by multiple environmental factors; yet, the high rates we observed likely indicate real trends. The cohort consists of extremely preterm infants enrolled in a clinical trial, so findings may not generalize to less preterm infants or different settings. Finally, while we identified associations with male sex, the biologic underpinnings require further elucidation beyond the scope of this study.

In summary, we report that male sex is associated with worse short and long-term renal outcomes in extremely preterm infants. These findings reinforce the concept that sex is an important biologic variable in neonates, and emphasizes the need to incorporate sex-disaggregated analyses in neonatal trials and observational studies [29]. Future work should focus on the mechanisms driving these disparities — for example, sex-specific differences in kidney development, hormonal influences, or immunologic responses to kidney injury. Given the modest effect size observed, our findings do not support altering existing monitoring practices based solely on biologic sex. However, sex may serve as one of multiple factors informing broader risk assessment in premature neonates. By acknowledging and investigating sex differences, we can better tailor care and improve long-term outcomes for these vulnerable children.

## Supplementary Information

Below is the link to the electronic supplementary material.

Graphical abstract (PPTX 527 KB)

## Data Availability

De‐identified individual participant data are available through the NINDS Data Archive: https://www.ninds.nih.gov/Current-Research/Research-Funded-NINDS/Clinical-Research/Archived-Clinical-Research-Datasets. The data includes de‐identified data with data dictionaries, in addition to study protocol, the statistical analysis plan, and the informed consent form.

## Declarations

### Prior presentation of study data

Presented as a poster at the 2024 International Neonatal Nephrology Symposium.
