# Supplementary material for: Examining the role of biologic sex on kidney outcomes in preterm neonates: A secondary analysis of the PENUT/REPAIReD study
Source: Pediatr Nephrol. 2026 Mar 12;41(8):2565–73. doi: 10.1007/s00467-025-07131-3 (PMC13337948; doi:10.1007/s00467-025-07131-3)
Supplement: Supplementary file 1 — Graphical abstract (PPTX 527 KB) [file 467_2025_7131_MOESM1_ESM.pptx]

## Slide 1
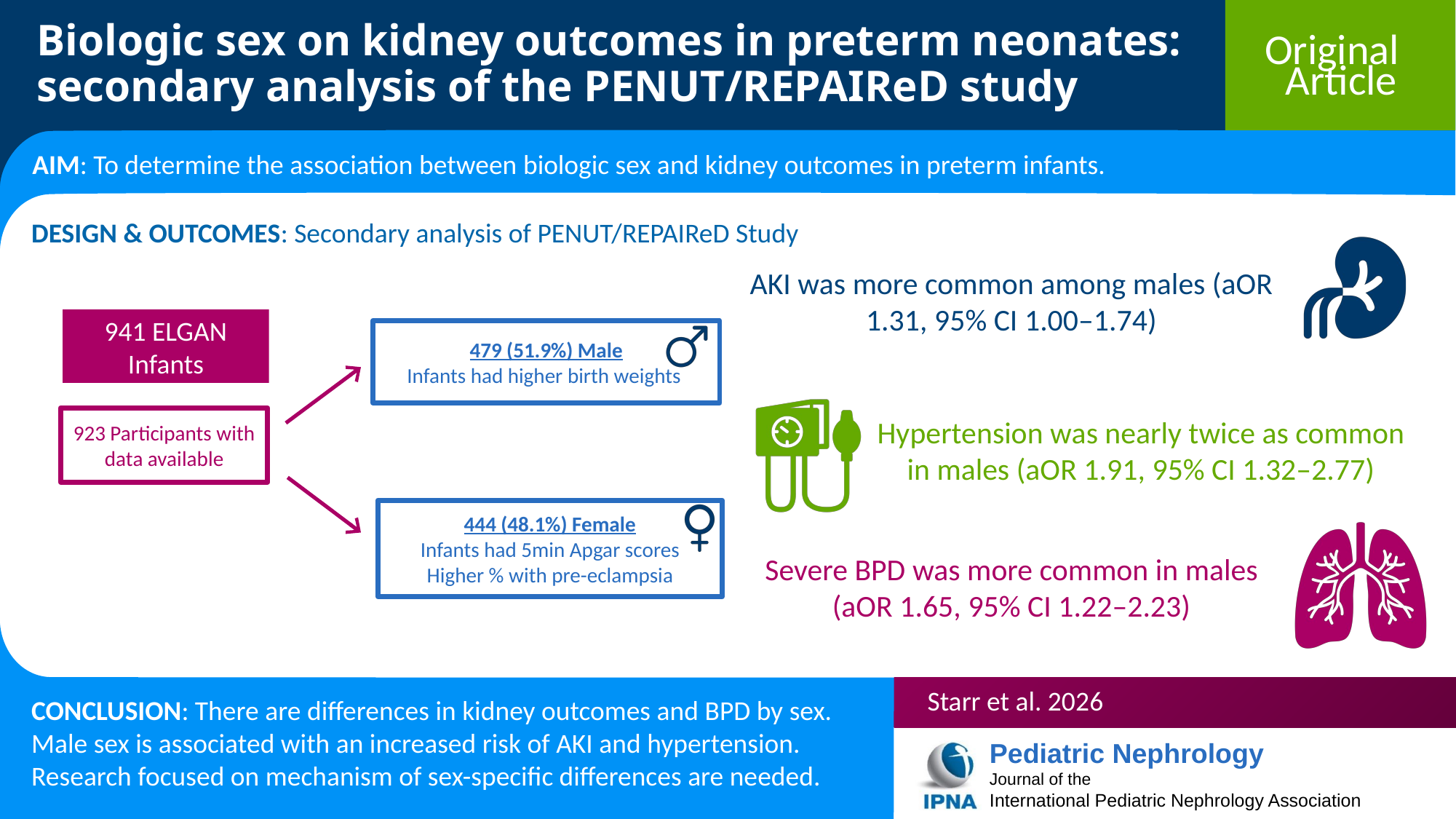

Biologic sex on kidney outcomes in preterm neonates: secondary analysis of the PENUT/REPAIReD study
AIM: To determine the association between biologic sex and kidney outcomes in preterm infants.
DESIGN & OUTCOMES: Secondary analysis of PENUT/REPAIReD Study
AKI was more common among males (aOR 1.31, 95% CI 1.00–1.74)
941 ELGAN Infants
479 (51.9%) Male
Infants had higher birth weights
923 Participants with data available
Hypertension was nearly twice as common in males (aOR 1.91, 95% CI 1.32–2.77)
444 (48.1%) Female
Infants had 5min Apgar scores
Higher % with pre-eclampsia
Severe BPD was more common in males (aOR 1.65, 95% CI 1.22–2.23)
Starr et al. 2026
CONCLUSION: There are differences in kidney outcomes and BPD by sex. Male sex is associated with an increased risk of AKI and hypertension. Research focused on mechanism of sex-specific differences are needed.
